# A Case of Bilateral Papilledema With Improved Clinical Symptoms by Venous Stenting for Superior Sagittal Sinus Stenosis

**DOI:** 10.7759/cureus.43828

**Published:** 2023-08-20

**Authors:** Masaki Miyoshi, Akio Tabuchi, Osamu Mimura, Atsufumi Nagahama, Hitoshi Tabuchi

**Affiliations:** 1 Ophthalmology, Tsukazaki Hospital, Himeji, JPN; 2 Ophthalmology, Saneikai Tsukazaki Hospital, Himeji, JPN; 3 Ophthalmology, Hyogo College of Medicine, Nishinomiya, JPN; 4 Neurosurgery, Tsukazaki Hospital, Himeji, JPN; 5 Ophthalmology, Hiroshima University, Hiroshima, JPN

**Keywords:** venous stenting, intracranial hypertension, papilledema, sss, superior sagittal sinus stenosis

## Abstract

Superior sagittal sinus (SSS) obstruction causes intracranial hypertension, often requiring surgical stenting. Consensus on treating brain venous sinus stenosis, another cause, is lacking. This study reports a case of SSS stenosis and intracranial hypertension treated with venous stenting, improving bilateral papilledema. A 51-year-old with a headache and visual disturbance had papilledema and visual field loss. MR venography showed SSS stenosis, leading to a neurosurgery referral. Lumbar puncture confirmed intracranial hypertension (>35 cmH2O), prompting venous stenting. Post-procedure, papilledema, headache, and visual field loss improved. Venous stenting could be effective for SSS stenosis with clinically proven or recurrent pressure differences. Further cases are needed for standardization.

## Introduction

Cerebral venous thrombosis (CVT) is a rare disease, accounting for less than 1% of all cerebral stroke cases [1]. It is a critical factor in causing strokes in young people. The mean onset age is 33 years, and the prevalence in women is thrice than that in men, suggesting the involvement of risks related to pregnancy and birth control pills [2]. If diagnosed and treated at an early stage, CVT often exhibits a favorable prognosis. A typical acute-phase treatment is an anticoagulant therapy mediated by heparin administration. However, if symptoms are aggravated regardless of treatment, intravascular treatment (stent placement), venous thrombectomy, and/or brain surgery (intracranial decompression) may be considered [3]. The most frequent clinical symptom is headache, which is noted in 90% of patients. However, this is not a specific symptom, as some patients do not develop any headaches [4]. While neurological focal symptoms are observed in 40%, they do not usually occur suddenly, unlike arterial infarction. It is often difficult to compare thrombotic sites with adjacent brain tissue with standard MRI and CT. In such cases, a thrombotic obstruction in a venous sinus or a cerebral vein needs to be detected by MR venography and/or CT venography [5]. No diagnosis may be reached if an obstruction or stenosis is incomplete, hence possibly contributing to idiopathic intracranial hypertension (IIH) [6,7].

Meanwhile, IIH was defined by Quincke in 1893 as intracranial hypertension not accompanied by a brain tumor [8]. It is a pathological condition that may cause headache, cranial nerve disorder, and papillary stasis papilledema due to increased intracranial pressure despite the absence of space-occupying lesions [9,10]. Although papilledema may either be asymptomatic or only exhibit an enlargement of Mariotte’s blind spot at an early stage, it aggravates vision/visual field impairment as optic atrophy progresses over an extended period [11].

This article reports a recently encountered case of SSS stenosis with few clinical symptoms, in which the long-term progress of papilledema and headache partially contributed to the diagnosis and treatment of intracranial hypertension.

## Case presentation

**Figure 1:**
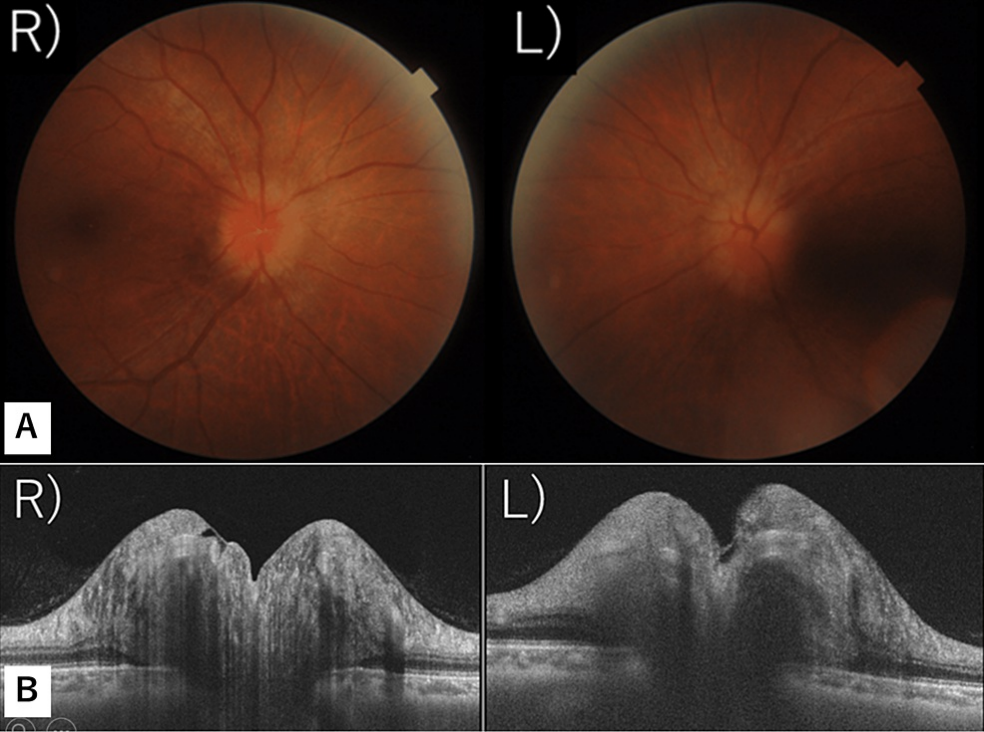
Papillary findings and papillary OCT findings at the first examination A. A color photograph of the posterior fundi: Prominent reddening of optic papillae is noted in both eyes, with the borders being irregular. B. Papillary OCT images: Prominent swelling of optic papillae is noted in both eyes.

**Figure 2:**
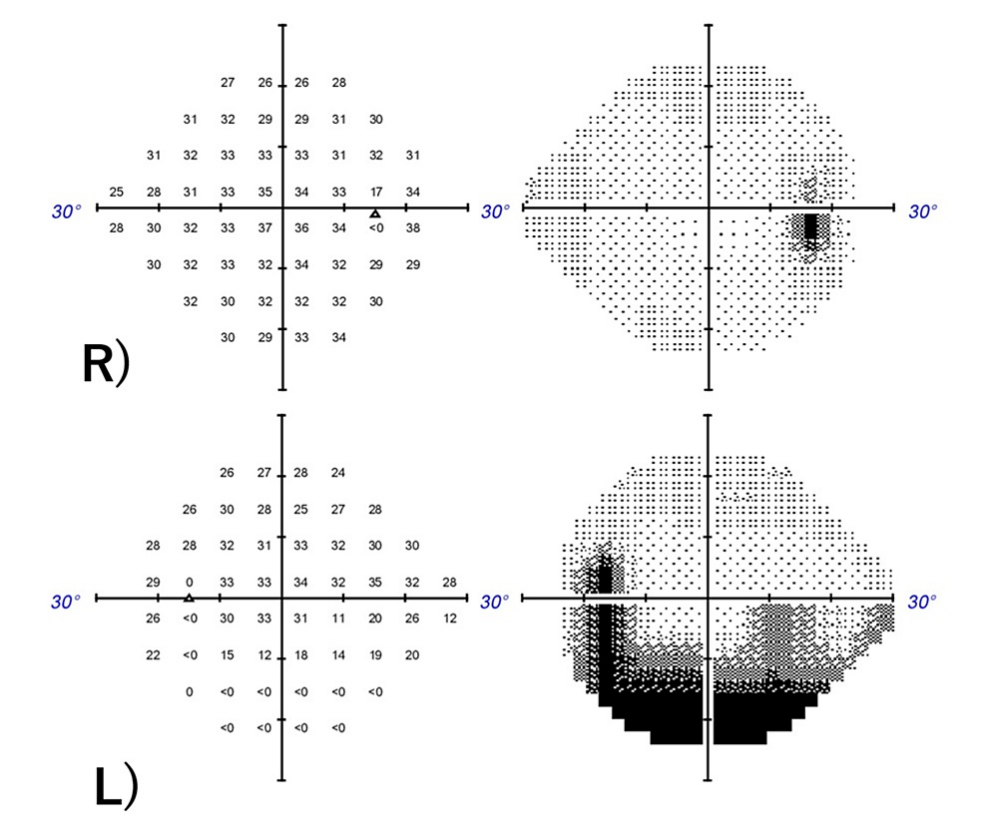
Humphrey visual field (30-2 program) at first examination Right eye (top), Left eye (bottom) Static visual field test: A hemianopia-like visual field defect was noted in the lower left eye.

A 51-year-old man presented with blurred vision, visual field impairment in the left eye, and an occipital headache that he had been experiencing for two weeks. His medical history was unremarkable. On his first visit, he had no painful eye movements. His intraocular pressure was 18 mmHg in both eyes. Ocular alignment was normal, with no limited eye movements or abnormalities in the anterior eye or optic media. However, there was prominent bilateral papilledema (Figures 1a, 1b). In both eyes, the light reflex was rapid and sufficient, and the relative afferent pupillary defect (RAPD) was negative. The critical flicker fusion frequency was measured as R: 35-38Hz and L: 37-38Hz. A Humphry visual field test showed inferior visual field impairment in the left eye and a slight change in the lower part of the right eye (Figure 2). Optical coherence tomography (OCT) did not reveal any thinning of the retinal ganglion cell layer in the macular areas of both eyes.

**Figure 3:**
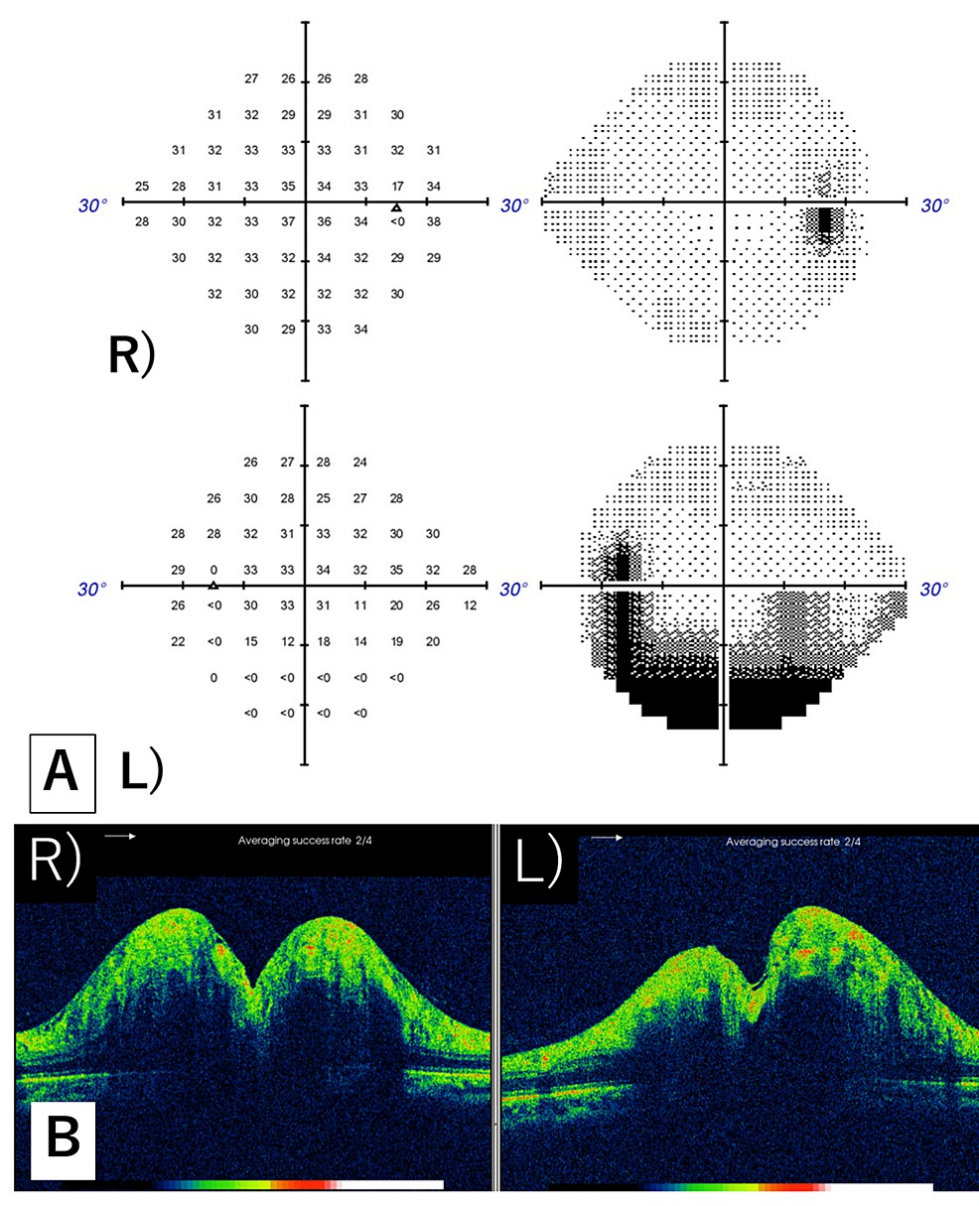
Humphrey visual field and papillary OCT findings three weeks after starting steroid pulse therapy A. Static visual field test: The visual field defect in the left eye slightly improved. B. Papillary OCT images: The mild swelling in both eyes improved.

**Figure 4:**
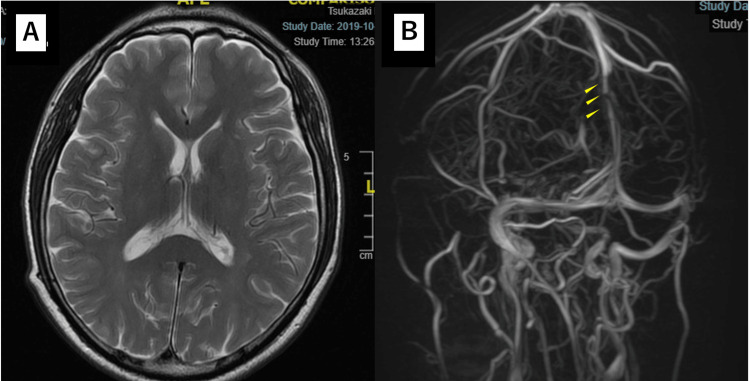
Cranial MRI and MRV A. MRI T2 FLAIR image: Neither intracranial space-occupying lesions nor cerebral ventriculomegaly were observed. B. MR venography: The arrows indicate superior sagittal sinus stenosis sites

**Figure 5:**
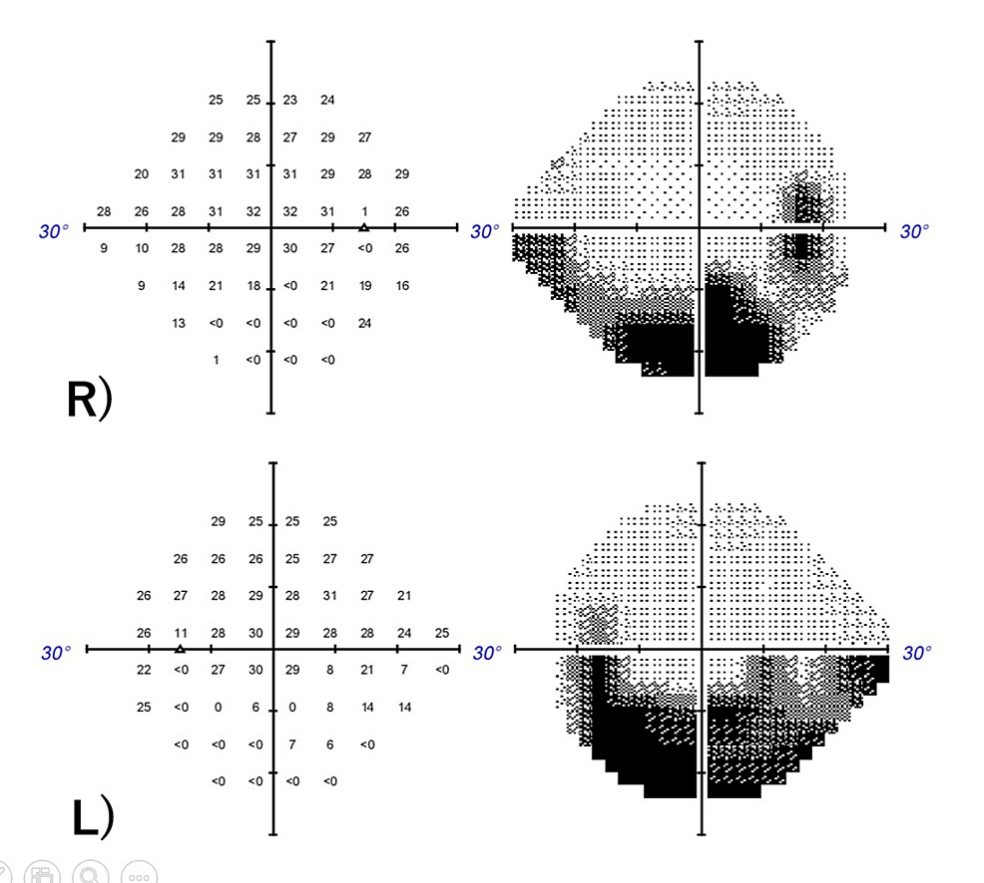
Six months after the first examination Right eye (top), Left eye (bottom) Static visual field test: A visual field defect was also noted in the lower right eye.

**Figure 6:**
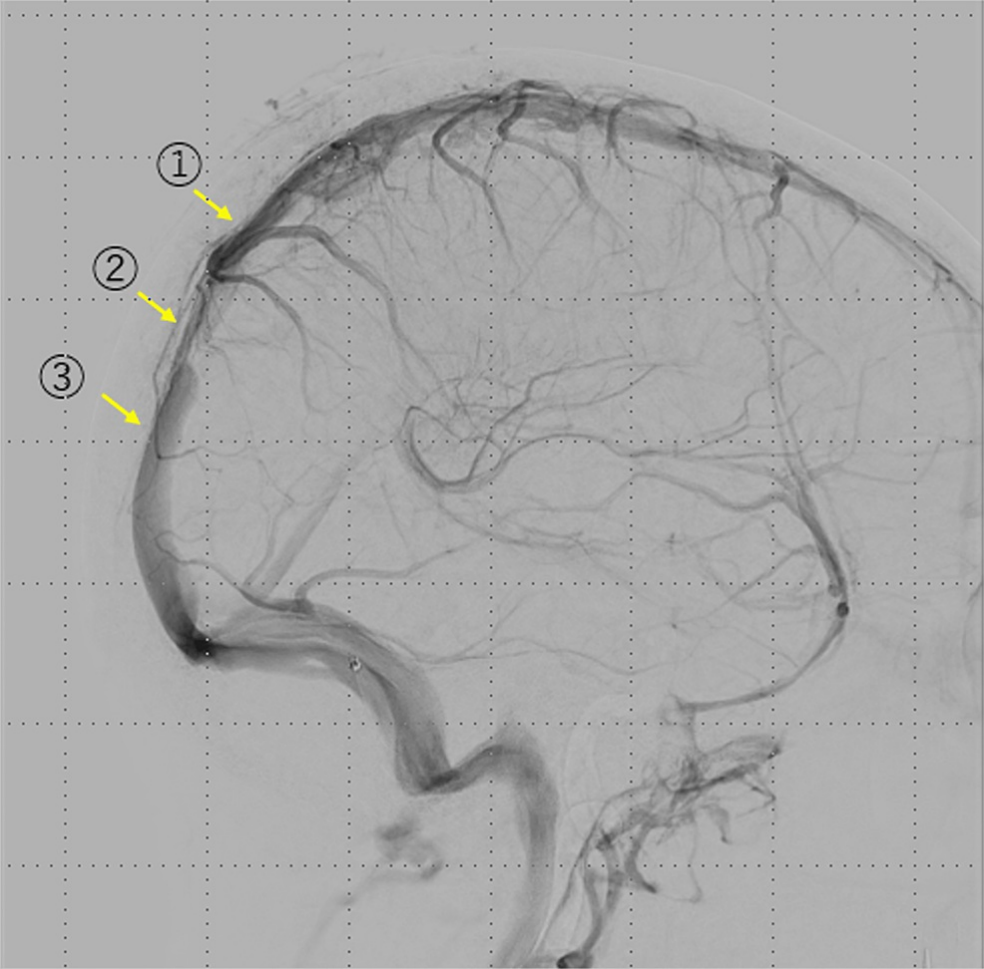
Photography of the left internal carotid artery, a venous-phase lateral image (1) Proximal stenosis site, (2) narrowest stenosis site, and (3) distal stenosis site.

**Figure 7:**
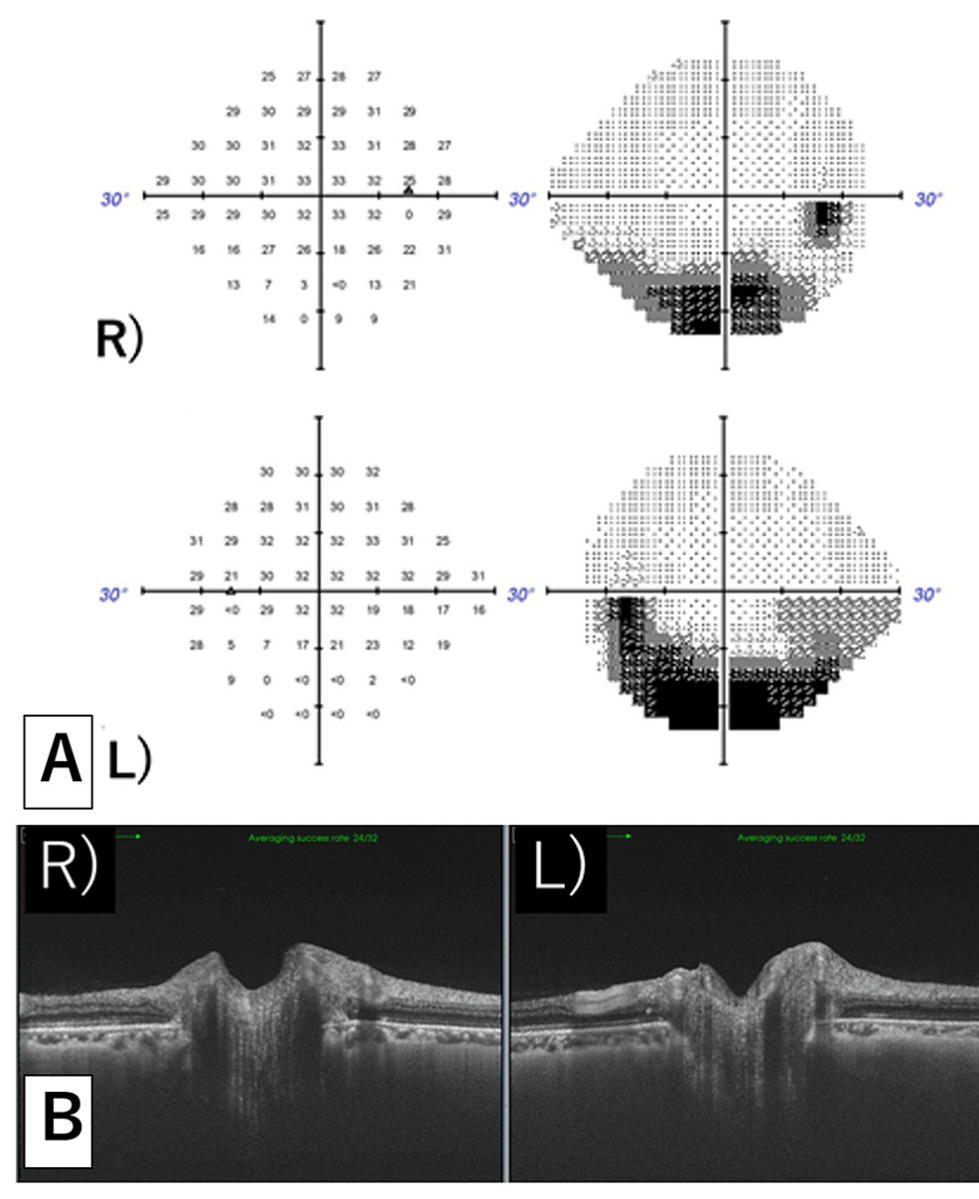
After stent placement A. Static visual field test: Changes in the visual field improved in both eyes, albeit with partial remnants. B. Optic papillary OCT: Swelling in both eyes apparently improved

On the day of the first examination, the patient was referred to the neurology department and underwent a cranial CT and MRI, which did not show any space-occupying lesions in the cranium (Figures 3a, 3b). Suspecting left optic neuritis, three courses of steroid pulse therapy were given (methylprednisolone 1000 mg for three days, followed by four days without the drug). This treatment led to an improvement in the left eye's vision and relief from the occipital headache, but the bilateral papillary swelling persisted. Magnetic resonance venography (MRV) indicated irregularities in the SSS wall, so the patient was further evaluated in the cerebral neurology department but did not receive treatment as there were no venous sinus thrombi (Figures 4a, 4b). Six months later, a hemianopsia defect appeared in the lower left eye, and changes in the visual field were noted in the right eye (Figure 5). The patient was referred to the cerebral neurosurgery department for an evaluation of intracranial hypertension. A lumbar puncture showed increased intracranial pressure above 35 cmH2O. After the lumbar puncture, the occipital headache and blurred vision temporarily improved. Venous pressure was measured both upstream and downstream of the SSS stenosis site, and stent placement was performed once pressure differences were confirmed (Figure 6). Before stent placement, venous pressure was 26/21 mmHg at the proximal stenosis site, 21/14 mmHg at the narrowest stenosis site, and 10/9 mmHg at the distal stenosis site. After stent placement, the pressures were 21/14 mmHg, 21/14 mmHg, and 20/14 mmHg, respectively. This considerably improved the papilledema in both eyes and slightly improved changes in the visual field in both eyes (Figures 7a, 7b).

## Discussion

When bilateral papilledema not accompanied by neurological abnormalities is noted, idiopathic intracranial hypertension is one of the diseases that should be presumed. When it is suspected, either cerebral neurological or neurosurgical therapeutic approaches should be prioritized. Recommended conservative management approaches include reducing body weight, reducing salt intake from meals, ingesting diuretic drugs, and improving lifestyle habits. If these prove ineffective, surgical approaches, including optic nerve sheath fenestration (ONSF), cerebrospinal fluid (CSF) shunt operation, and stent placement, are performed [6,12].

Thrombi may develop in various outflow channels of cerebral veins, with the most frequent sites including the SSS transverse sinus, cavernous sinus, and sigmoid sinus. The most frequent site is the SSS, which accounts for approximately 60% of cerebral venous outflow obstruction sites [13].

Obstruction in the SSS is widely known to increase intracranial pressure and cause papilledema. However, the site was not completely obstructed in this patient, thus rendering it difficult to reach a definite preoperative diagnosis of whether the persistent papilledema was caused by SSS stenosis. Other than the bilateral papilledema and the occipital headache, no apparent neurologically abnormal findings were noted, and MRI (T1, T2, and DWI) and MRV only detected wall irregularities in the SSS. After the lumbar puncture performed at the cerebral neurosurgery department confirmed an increase in the opening pressure, a diagnosis of intracranial hypertension was reached. Given the approval of the ethics committee, pressure differences between upstream and downstream of the stenosis sites were checked using a cerebrovascular catheter. In this case, stent placement treatment was selected, and it was effective.

Previous studies have revealed that cerebral sinus stenosis is a factor causing IIH [6,7], with MRV detecting sinus stenosis in 30%-93% of all IIH patients [14]. However, only a few reports have stated that resolving pressure differences through stent replacement improved symptoms. Hence, no standardized therapeutic guidelines are available on when to perform intravenous stent placement as part of IIH treatment [15].

If papilledema persists over an extended period, irreversible impairment in vision/visual field may occur [16,17]. In this case, approximately a year had passed since the first examination until the patient was diagnosed and treated. During this period, changes in the visual field progressed along the optic nerve fibers. Although the patient achieved an improvement in the visual field impairment after the treatment, the changes in the visual field might have remained without complete recovery because the symptoms progressed over an extended period.

## Conclusions

In this study, pressure differences were observed upstream and downstream of the cerebral sinus stenosis sites. This result suggests that stent placement is an effective and viable choice for cerebral sinus stenosis. Further accumulation of similar case study data is desired.
